# Highly Sensitive Detection of Chemically Modified Thio-Organophosphates by an Enzymatic Biosensing Device: An Automated Robotic Approach

**DOI:** 10.3390/s20051365

**Published:** 2020-03-02

**Authors:** Giovanni Paolo Cetrangolo, Janis Rusko, Carla Gori, Paola Carullo, Giuseppe Manco, Marco Chino, Ferdinando Febbraio

**Affiliations:** 1 Institute of Biochemistry and Cell Biology, National Research Council (CNR), Via P. Castellino 111, 80131 Naples, Italy; g.cetrangolo@ibp.cnr.it (G.P.C.); janis.rusko@bior.lv (J.R.); c.gori@ibp.cnr.it (C.G.); p.carullo@ibp.cnr.it (P.C.); 2 Institute of Food Safety, Animal Health and Environment “BIOR”, Lejupes Street 3, LV-1076 Riga, Latvia; 3 Department of Chemical Sciences, University of Naples “Federico II”. Via Cintia, 80126 Napoli, Italy; marco.chino@unina.it

**Keywords:** esterase, biosensing device, *Alicyclobacillus acidocaldarius*, thio-organophosphate, fluorescence assay, robotic workstation

## Abstract

Pesticides represent some of the most common man-made chemicals in the world. Despite their unquestionable utility in the agricultural field and in the prevention of pest infestation in public areas of cities, pesticides and their biotransformation products are toxic to the environment and hazardous to human health. Esterase-based biosensors represent a viable alternative to the expensive and time-consuming systems currently used for their detection. In this work, we used the esterase-2 from *Alicyclobacillus acidocaldarius* as bioreceptor for a biosensing device based on an automated robotic approach. Coupling the robotic system with a fluorescence inhibition assay, in only 30 s of enzymatic assay, we accomplished the detection limit of 10 pmol for 11 chemically oxidized thio-organophosphates in solution. In addition, we observed differences in the shape of the inhibition curves determined measuring the decrease of esterase-2 residual activity over time. These differences could be used for the characterization and identification of thio-organophosphate pesticides, leading to a pseudo fingerprinting for each of these compounds. This research represents a starting point to develop technologies for automated screening of toxic compounds in samples from industrial sectors, such as the food industry, and for environmental monitoring.

## 1. Introduction

This century has been marked by the replacement of environmentally persistent organochlorine pesticides by the less persistent organophosphate (OP) and carbamate pesticides. However, OPs have become the most widely used chemicals with well-known neurotoxic effects [1,2,3,4,5]. These compounds were designed to bind with high affinity to the active site of acetylcholinesterase in order to selectively inhibit these enzymes in insect pests. Unfortunately, the biochemical mechanisms underlying the functions of the brain between insects and humans are very similar, and these substances inevitably end up affecting human health [1]. Several authors have described a possible relationship between pesticide exposure and neurodevelopmental disorders [2]. In particular, children are more sensitive to exposure to neurotoxic compounds as their brains are in a stage of rapid development and the ratio of pesticide-dose/body-weight is higher than that in adults. Moreover, the physiological detoxification systems of children are less efficient than those of adults [2,5,6,7]. Facing the impossibility of banning the use of pesticides in agriculture and the awareness of their presence in foods and in the environment (water, soil, etc), due to environmental factors such as rain and wind, action for the detection and monitoring of these compounds is mandatory [8]. Conventional analytical techniques, such as gas and liquid chromatography combined with mass spectrometry measurements [9,10], are powerful tools for OP detection. However, some limitations in their use should be considered, such as, the high-cost of operation, long time required for the analysis, the requirement of skilled labour and sometimes the unsuitableness for in situ and real-time detection. Fortunately, the development of new, selective and sensitive sensors allows for increasingly easy and cheap ways to monitor and detect toxic and hazardous substances in the environment [11,12,13,14], possibly also permitting individual citizens in the near future, to screen for different contaminants. Several biosensors for OP detection based on enzymatic or biological (cells and microorganisms) bioreceptors have been previously proposed [15,16,17]. The most known are biosensors based on the inhibition of the activity of acetylcholinesterase [17,18], which is the target of Ops’ mechanism of action. However, the use of these enzymes has several limitations, such as the low stability of these proteins and their susceptibility to react with a wide number of compounds other than OPs. Thus, it has become important to search for new and more efficient enzymatic activities to use as bioreceptors for these types of biosensors. In our previous work [19], we were able to determine via in vitro inhibition assays the inhibition of esterase-2 from *Alicyclobacillus acidocaldarius* (EST2) using paraoxon, with good reproducibility and sensitivity. EST2 is a thermostable, single chain protein of 34 kDa molecular weight, belonging to the hormone-sensitive lipase family. Its overall structural fold is typical of α/β hydrolases, with an active pocket possessing a lipase-like Ser-His-Asp catalytic triad [20]. The enzyme showed a very high affinity toward paraoxon [21], producing a stable covalent complex irreversibly inactivating the enzyme. In addition, EST2 showed a better selectivity than the non-specific reactions of acetylcholinesterase, the main target of OP pesticides [19]. The experiments carried out allowed the detection of very low amount of neurotoxic agent, reaching a quantification limit of 125 fmol, comparable to the efficiency of more widely used analytical systems [22]. Moreover, EST2 activity was maintained in presence of complex matrices, such as fruit juices or urine [19,22]. In fact, in previous works we already evaluated the possibility to perform activity assays after enzyme inhibition with aliquots of fruit juices deliberately contaminated with paraoxon [19]. This interesting feature would allow the enzyme use in real conditions on untreated samples. In addition, the EST2 long-term stability, combined with the ability to immobilize the enzyme on nitrocellulose membranes without affecting the enzymatic properties [19] made EST2 a special candidate for the design of a biosensor for OP detection.

In the present paper, we have focused on two specific aspects in OP detection using EST2. Firstly, we investigated the possibility to increase the number of detectable OPs by chemical oxidation of phosphorothionate compounds [23,24]. In fact, the phosphorothionate OPs are generally poor inhibitors of cholinesterases [25], including EST2 [19], requiring to be activated by biotic transformation processes (microorganisms or plants) and/or by abiotic processes, such as chemical and photochemical reactions. Secondly, we combined the use of fluorescence measurements for the residual enzymatic activity with a robotic approach, in order to develop a novel screening methodology, suitable for large-scale monitoring analysis.

## 2. Materials and Methods

### 2.1. Reagents

All reagents were of analytical grade and obtained from commercial sources. 2-[4-(2 Hydroxyethyl)-1-piperazino]ethansulfonic acid (HEPES), 4-methylumbelliferyl butyrate (4-MUBu), 4-methylumbelliferone (4-MU), N-bromosuccinimide (NBS), diethyl (4-nitrophenyl)phosphate (paraoxon), diethoxy-(4-nitrophenoxy)sulfanylidenephosphorane (parathion), 3-chloro-7-diethoxy-phosphinothioyloxy-4-methyl-chromen-2-one (coumaphos), dimethoxy-(4-nitrophenoxy)-sulfanylidenephosphorane (methyl parathion), diethoxy-(4-methylsulfinylphenoxy)-sulfanylidene-phosphorane (fensulfothion), O-4-cyanophenyl O,O-dimethyl phosphorothioate (cyanophos), diethoxysulfanylidene-(3,5,6-trichloropyridin-2-yl)oxyphosphorane (chlorpyrifos), diethoxy-(6-methyl-2-propan-2-ylpyrimidin-4-yl)oxy-sulfanylidene-phosphorane (diazinon), N-(mercapto-methyl)phthalimide S-(O,O-dimethyl) phosphorothionate (phosmet), O-(2-(diethylamino)-6-methyl-4-pyrimidinyl) O,O-dimethylphosphorothioate (pirimiphos), O,O-dimethyl O-(2,6-dichloro-4-methylphenyl) phosphorothioate (tolclofos), were from Sigma-Aldrich (St. Louis, MO, USA).

### 2.2. Enzyme Purification

EST2 was overexpressed in the mesophilic host *E. coli* strain BL21 (DE3) and purified as previously described in Manco et al. [26]. Purity was tested by SDS-PAGE. The protein concentration was estimated by the optical absorbance at 280 nm, using a molar extinction coefficient of 1.34 × 10^5^ M^−1^ cm^−1^ in 40 mM sodium phosphate buffer, pH 7.1, at 25 °C, as described in Manco et al. [26].

### 2.3. Fluorescence Standard Enzymatic Assay

The standard assay was prepared as previously described in Cetrangolo et al. [22]. Briefly, aliquots of EST2 were assayed at 30 °C in a volume reaction of 0.5 mL, containing 25 mM HEPES buffer, pH 7.0, 1% Triton X100 and 1 mM 4-MUBu (from a stock solution of 40 mM 4-MUBu in 100% DMSO), using a quartz cuvette of 1 cm optical path. Fluorescence measurements were carried out by monitoring the increase of fluorescence emission at 445 nm (Ex = 365 nm) due to the release of 4-MU as reaction product, using a JASCO FP-777 spectrofluorimeter (Jasco Analytical Instruments, Tokyo, Japan), equipped by an external thermostatic bath (F25, Julabo, Seelbach, Germany). One unit of enzymatic activity was defined as the amount of enzyme required to release 1 μmol/min of 4-MU, determined using a coefficient value of 57.16 ± 1.03 fluorescence units/pmol of 4-MU, as described in Cetrangolo et al. [22].

### 2.4. Inhibition Assay of EST2 in Presence of Pesticides

Ten mM stocks of paraoxon, of coumaphos, of fensulfothion, of methyl-parathion, of parathion, of cyanophos, of pirimiphos and of diazinon in 100% DMSO, and 20 mM stocks of phosmet, of chlorpyrifos and of tolclofos in 100% DMSO, were prepared in order to use as EST2 activity inhibitors. The inhibition assays were carried out under the standard assay conditions by incubating aliquots of 1.46 pmol of EST2 in presence of increasing concentrations of each inhibitor in the range from 0 to 2.1 pmol in a final volume of 10 μL. After 1 min incubation, aliquots of inhibited enzyme were removed from the mixture and the residual activity was measured in the standard assay conditions. The inhibition percentage was calculated based on the following as previous described [27] equation:

(I_0_ − I)/I_0_ × 100 (1)

in which I_0_ represent the inhibition percentage in absence of inhibitor and I the percentage of inhibition at the indicated inhibitor concentration. All measures were carried out at least three times and the data analysed by the software QtiPlot 0.9.8.9 (Copyright 2004–2011 Ion Vasilief).

### 2.5. Phosphorothionate Pesticide Oxidation by NBS

The chemical oxidation of phosphorothionate compounds was carried out by incubating these compounds in the presence of NBS. Aliquots of NBS (90 mM in water) were added at the final concentration of 300 μM to aliquots of OPs which possess the sulphur atom in binding with phosphate, at room temperature, in a ratio 1:90, and immediately mixed. After 5 min of incubation, the mixture was used in the inhibition assays of EST2. Incubation time and concentration ratio were determined by measuring the inhibition efficiency of oxidized-parathion on EST2 activity and the lack of NBS effects in the enzymatic activity.

### 2.6. MS Analysis of NBS-Oxidised Pesticides

The chemical oxidation of phosphorothionate compounds by NBS was tested by using LC-MS approach. LC was performed with a Nexera X2 series UPLC system (Shimadzu, Kyoto, Japan). The chromatographic separation was achieved on a Symmetry C_18_ column (5 µm, 4.6 × 150 mm, Waters, Dublin, Ireland). The column oven temperature was kept at 40 °C and the temperature of autosampler was set at 10 °C. Flow rate was set at 0.400 mL/min. Compounds were separated with a gradient of water (A) and ACN (B), both with added 4 mM ammonium acetate and 0.1% acetic acid. Gradient conditions were as follows: 5 min equilibration at initial 5% B, then increased to 60% B at 2 min with a further increase to 90% B at 5 min, followed by final increase to 95% B at 12 min. Gradient was returned to initial conditions of 5% B during 0.5 min and allowed for 2.5 min re-equilibration. Total analysis time was 20 min. Injection volume was set to 10 µL. MS analysis was performed with a 4500 QTrap mass spectrometer (AB Sciex, Courtaboeuf, France), equipped with turbo ion spray interface (ESI), operated in positive ionization mode. The source conditions were as follows: ion spray voltage, 5.5 KV; curtain gas pressure, 25 psi; nebuliser and heating gas pressure, 50 psi. The source temperature was set at 500 °C. Data acquisition was performed with the MRM mode. The declustering potential, the collision energy and the cell exit potential were found from literature data. Detailed MRM transitions and voltage settings are shown in Supplementary Table S1. Data were plotted by using the software QtiPlot 0.9.8.9.

### 2.7. Determination of EST2 Activity in Presence of NBS

The effect of NBS on the activity of EST2 was measured by pre-incubating 1.46 pmol of EST2 in the presence of several concentrations of NBS in the range from 0 to 50 mM in a final volume of 10 μL. After 1 min of incubation, aliquots of EST2 were taken from the mixture and the residual activity was measured in the standard assay conditions. All measures were carried out at least in triplicate and the data analysed by the software QtiPlot 0.9.8.9.

### 2.8. EST2 Assay on Robotic Workstation and OP Screening

**Figure 1:**
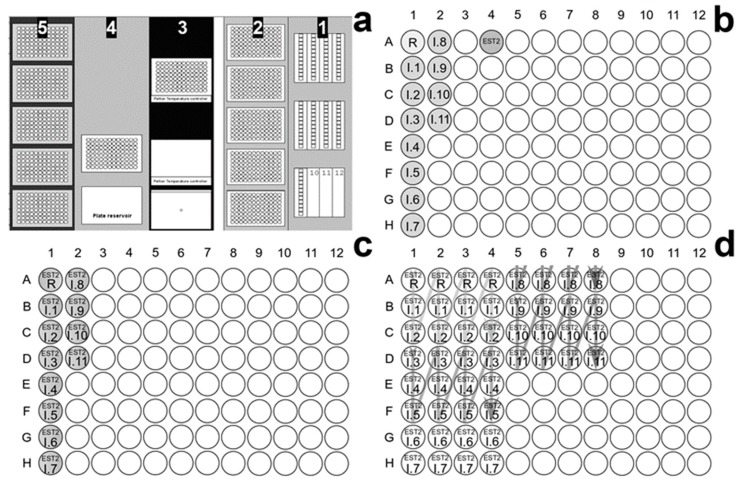
Representation of the robotic workstation worktable and plates layout for the measurement of EST2 residual activity. (**a**) In the worktable column 1 the solvent reservoir carriers, in column 2 disposable tip carriers (10, 200, 1000 μL), in column 3 the stock solution and reagent plate in the temperature controller, in column 4 the incubation plate on the heated shaker, and in column 5 the plates for the enzymatic residual activity assays, were placed. (**b**) Plate placed in column 3 of (**a**), prepared by the operator manually, containing the enzyme (EST2) and the NBS treated OP inhibitors (I.1, I.2, … I.n) and a blank reference (R). (**c**) Plate in column 4 of (**a**), containing the incubation mixtures (enzyme-inhibitors) prepared by the robotic liquid handler. (**d**) Plate layout in column 5 for the assays of enzymatic residual activity, samples are divided in three groups for the assay of each mixture. The arrows indicated the direction of measurements for each group of assays.

Enzyme activity assay in the presence of irreversible inhibitors for pesticide screening and fingerprint purpose was carried out in 96 well micro-plates V-bottom, black, from Greiner Bio-one (Kremsmünster, Austria), using a Microlab^®^ STAR Liquid Handling Workstation (Hamilton Europe, Bonaduz, Switzerland), equipped with an eight-channel liquid handler arm and a Hamilton Microlab^®^ iSwap robotic arm, a gripper tool that can access plates on or off the deck, and Hamilton Heater Shaker. The STAR line workstation is equipped with a sensor for the control of temperature set in our experiments at 20 °C and controlled from a Microlab^®^ Star Vector software 4.0. The workstation was also connected to a VICTOR^TM^ X3 Multi-label Plate Reader (PerkinElmer, Waltham, MA, USA) a luminescence, fluorescence, and UV-Absorbance reader, equipped with a dispenser module and a shaker with adjustable speed. In Figure 1a, the layout of the robotic workstations worktable is described. In the worktable column 1 the first solvent reservoir carrier (total volume of about 250 mL) was filled with 50 mL of 0.025 M HEPES buffer pH 7.0, and 96-well micro-plates were placed in the worktable columns 3, 4 and 5. As described in Figure 1b, the mother plate in the temperature controller (set at 20 °C) of column 3 in Figure 1a, was prepared manually by the operator to contain 150 μL of EST2 enzyme at the final concentration of 0.7 μM in 0.025 M buffer HEPES pH 7.0 in the well 4A, and 11 different NBS treated OP inhibitors (10 μL each) at a final concentration of 3.3 μM in 3.3% DMSO, pre-incubated in presence of 300 μM NBS, in the wells from 1B to 2D, and a blank reference sample containing 3.3% DMSO and 300 μM NBS in water in the well 1A.

Incubation mixtures were prepared by the robotic liquid handler in the plate of worktable column 4, using the solutions from the solvent reservoir in column 1 and the plate in column 3, and dispensing in the wells from 1A to 2D the following solutions: 187 μL of 0.025 M HEPES buffer pH 7.0, 10 μL of EST2 (7 pmol). Then 3 μL of reference sample in the well 1A and 3 μL of each inhibitor (9.9 pmol) in the wells from 1B to 2D (Figure 1c). The plate was agitated in the shaker for 5 s and 10 μL aliquots, containing 0.35 pmol of fully inhibited enzyme with 0.495 pmol of the inhibitors, were withdrawn and dispensed in the plates in the worktable column 5 (each mixture had four replicates) for the assay of enzymatic residual activity (Figure 1d). The Microlab^®^ iSwap robotic arm was used to transfer the plate to the Victor X3 plate reader, where the internal temperature was set at 30 °C, and the dispenser added to a single well 250 μL of a reaction mixture containing 25 μM MUBu in 0.025 M HEPES pH 7.0, 1% Triton X-100. Then the plate was shaken for 5 s and the increase in fluorescence at 445 nm, after excitation at 350 nm, was measured for 30 s. The instrument automatically was set to repeat the entire process of dispensation/agitation/reading for each well, until all the inhibitors were tested. All measures were carried out four times and the data analysed by the software QtiPlot 0.9.8.9.

## 3. Results and Discussion

### 3.1. In Vitro Pesticides Activation by Using NBS

**Figure 2:**
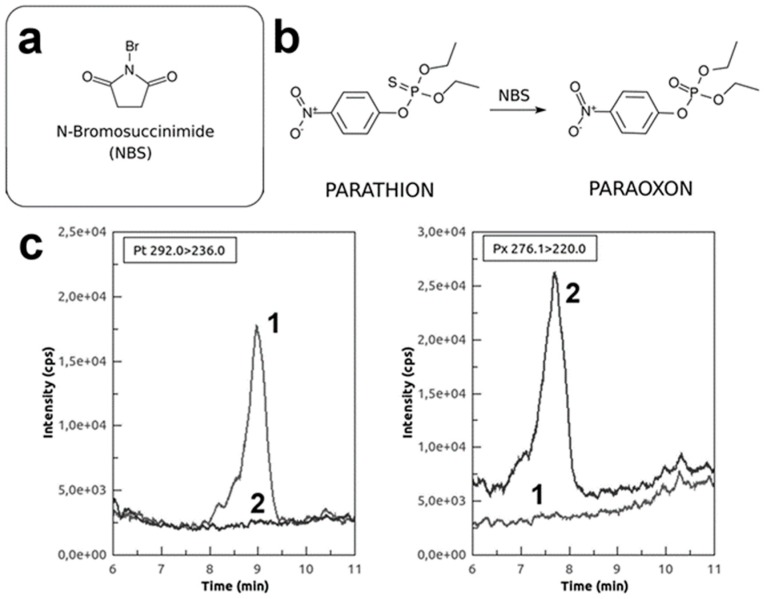
NBS-oxidized pesticides detection in water. (**a**) Chemical structures of N-bromosuccinimide and general formula of thio- and oxo-organophosphates. (**b**) NBS-mediated parathion/paraoxon transition. (**c**) LC-MS plot of transformation from parathion (baseline 1) to paraoxon (baseline 2), before (on the left) and after (on the right) the NBS oxidation step.

Considering the massive presence on the market of phosphorothionate OPs, we investigated the ability of EST2 to detect these compounds. EST2 similarly to acetylcholinesterase activities showed less affinity toward tio-OP, although it is able to reversibly bind many of these compounds such as parathion and chlorpyriphos [28,29]. Starting from the evidence that thio-OPs required to be activated in their active form of oxon-OP products, in order to irreversibly inhibit the acetylcholinesterase activities [30], we applied the same principle to the irreversible inhibition of EST2. We carried on inhibition assays experiments in combination with an oxidant pre-treatment of the thio-OP compounds using a selective and rapid oxidant agent such as NBS (Figure 2a,b), obtaining a complete conversion into oxons form within 5 min as described by Bavcon et al. [23]. The MS measurements of NBS treated thio-OPs, such as parathion, in a ratio 90:1 for 5 min, indicated the complete transition from thio-OP compounds to the oxon-OP counterparts (Figure 2c). In Figure 2c, the MS signal for parathion mass transition 292.0 -> 236.0 *m*/*z* (baseline 1) and for paraoxon mass transition 276.1 -> 220.0 *m*/*z* (baseline 2), were reported. Before the oxidation step, only parathion is present in the chromatogram and, as indicated by baseline, is it all converted to paraoxon after the oxidation.

### 3.2. Effects of NBS on EST2 Activity

Measurements of the EST2 residual activity in the presence of NBS indicated that the chemical modification reagent slightly influenced the enzyme activity. In particular, only 9.3 ± 1.9% of the EST2 activity was inhibited by 30 μM NBS (Supplementary Figure S1), corresponding to the final amount of oxidant in the inhibition assay of NBS-oxidized pesticides. These results are in agreement with data obtained with other esterase activities [31]. To avoid deviancy in results, all the measurements were carried out using a reference enzymatic activity of EST2 in both normal and oxidative (with addition of NBS) conditions.

### 3.3. EST2 Inhibition Measurements on in Vitro NBS-Activated Pesticides

The principle of EST2 inhibition by OPs was largely described in literature [19,21]. Briefly, the reactive organophosphorus group reacts with the catalytic serine residue in the active site of enzyme producing a covalent intermediate. This results in the irreversible inhibition of EST2 characterized by a progressive decrease of enzymatic activity over time, until complete inhibition in presence of inhibitor excess. The most interesting thing is that the affinity showed by EST2 toward the paraoxon is so high and the reaction so fast, that cannot be measured by the conventional method of pseudo first-order rate-constant for the determination of irreversible inhibition [19,32]. The high interest in bioreceptors showing high affinity irreversible inhibition, is particularly evident in the case of compounds that are highly toxic for living organisms, such as pesticides or nerve agents. By exploiting the fast inhibition rate of high affinity irreversible inhibitors, it becomes possible to measure the amount of toxic compound independently from the time and the amount of inhibitor, having as its only limit the detection of enzymatic activity. In fact, an increased sensitivity and reproducibility of the enzyme assay increases the possibility to measure small differences in the residual activity of the enzyme with increased sensitivity in the determination of the inhibitor concentrations. Using a fluorescence based assay, in our previous work we detected 230 fmol of paraoxon at 10% of inhibition, reaching a quantification limit of 125 fmol of pesticide [22]. We used the same conditions for the assays of thio-OP pesticides.

**Figure 3:**
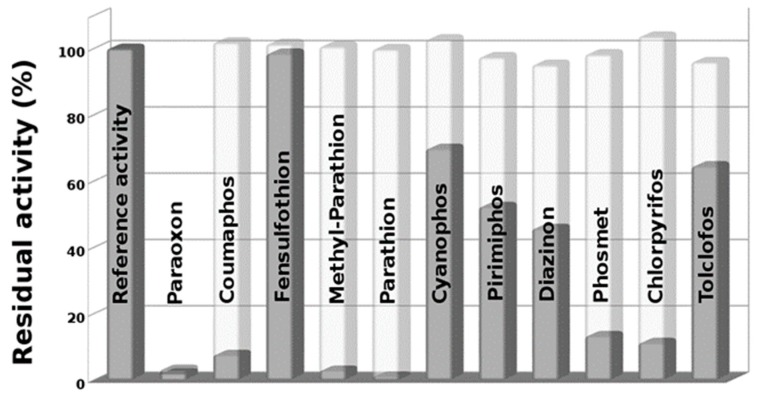
Plot of EST2 residual activity in absence (reference activity) and presence of different pesticides in their native form (light) and oxidized with NBS (dark).

The plotted data of the residual activity of EST2, with respect to the various pesticides tested (Figure 3) in both conditions—without and after NBS oxidation—revealed the capability of oxidized thio-OPs to irreversibly inhibit the EST2 activity. As expected, data indicated a complete loss in residual activity for methyl-parathion and parathion pesticides after NBS treatment. In fact, these thio-OP compounds, as demonstrated by mass spectrometry measurements on parathion (Figure 2c), are transformed in methyl paraoxon and paraoxon, respectively, which completely inhibit the EST2 activity in very short time [19,21]. Moreover, we observed a significant reduction of EST2 residual activity for chlorpyrifos, coumaphos and phosmet. Also, the oxidized form of diazinon, cyanophos, pirimiphos and tolclofos reduced the enzyme activity of about 30–50% with respect the same phosphorothionate compounds.

No significant effects were observed for the inhibition measured by assaying fensulfothion after oxidation. The pesticide paraoxon, as expected, showed a total reduction in the enzyme activity without oxidant pre-treatment in this experiment, as an excellent positive control.

We have already demonstrated the binding of some thio-OP pesticides, such as parathion, diazinon and chlorpyrifos [28], to the acyl- or alcohol-binding pockets of EST2. This means that EST2 is already able to bind almost all the OP compounds, including thio-OP. However, these intermediates were non-covalently bonded to the protein when in the thio-form, because of the low reactivity of sulphur group. Thus, in presence of an excess of colorimetric or fluorescent substrates for this enzyme, the pesticide is displaced from the catalytic site and the enzymatic activity remains unaltered. The obtained data is significant and clearly demonstrates the difference between the efficacy of inhibition for the pesticides tested in oxidizing conditions compared to the original ones. In fact, in the latter conditions the oxon-OP products irreversibly inhibited the EST2 activity as determined by the decrease in activity over time. Data also confirms the previously proposed hypothesis [33] that the replacement of sulphur with oxygen brings an increased positive charge density on the central phosphorous atom that allow for a more favourable nucleophilic attack of the -OH group of serine, irreversibly inhibiting the enzyme activity. The oxidative forms of tested compounds can be produced during storage and by breakdown products released from microorganisms or through natural exposition to light during application in the external environment. Our findings support the hypothesis that mandatory monitoring and analysis is necessary of oxidized forms of thio-OP for a complete risk assessment.

### 3.4. Automatic Approach in the Pesticide Determinations by Robotic System

In this study, we tried to develop an automatized assay allowing a streamlined, multi-sample analysis process using a biosensing device. Our goal was reached by exploiting an automatized approach on a Microlab^®^ STAR Liquid Handling Workstation equipped by a robotic arm and a VICTOR^TM^ X3 Multi-label Plate Reader. Using the robotic workstation, we were able to develop a protocol for a 96-well plate assay using EST2, the pesticide paraoxon as standard molecule and 4-MUBu as a substrate of the enzymatic reaction that can also provide the fluorescent signal. As a first step, we assessed the existence of a linear relationship between amount of 4-MU used and fluorescence intensity measured. The data obtained demonstrated a good linearity (R^2^ = 0.9975) between fluorescence intensity measured by the VICTOR^TM^ plate reader and 4-MU in the assayed range of concentrations (0.4–3.2 mM) (Supplementary Figure S2). The accuracy of robot for the dispensation of 4-MU aliquots in the plate wells was tested. In agreement with the standard assay for the spectrofluorometer, we observed similar results for the assay of EST2 activity using 4-MUBu as substrate in the plate reader by measuring a linear increase of the signal of fluorescence intensity. Using the fluorescence based assay, we obtained a sensitivity of 4.3 × 10^−2^ ± 2.2 × 10^−3^ in the linear range (R^2^ = 0.986) from 500 to 2000 fmol of paraoxon (Supplementary Figure S3), mathematically represented by the linear function y = mx, where x is the concentration of paraoxon, y is the residual activity, and m is the sensitivity of the biosensor. In these conditions, we calculated a LOD of 205.5 ± 6.98 fmol at 10% of inhibition and a precision within the 5% of standard deviation. The precision, as well as the accuracy in the determination of paraoxon, increases at higher pesticide concentration levels, because the ratio enzyme-inhibitor is closer to 1:1 ratio, as widely explained in our previous works [19,21].

**Figure 4:**
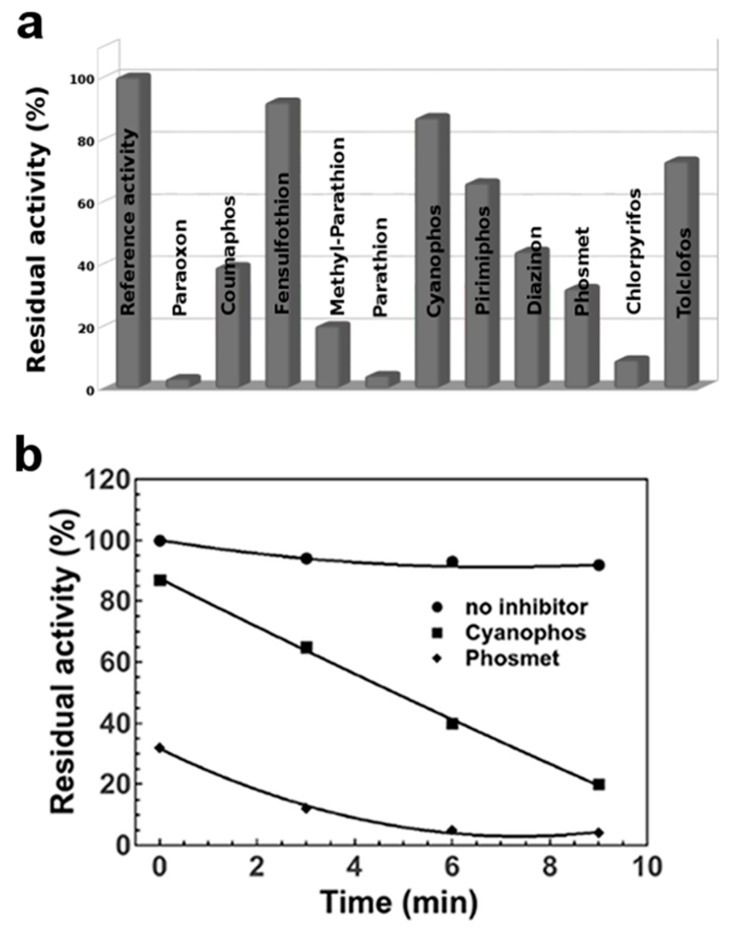
Robotic approach for OP detection in water. (**a**) Plot of EST2 residual activity in absence (Reference activity) and presence of different pesticides oxidized with NBS, measured using the robotic workstation. (**b**) Plot of EST2 residual activity measured using the robotic workstation at different inhibition times in absence and in presence of cyanophos and phosmet oxidized with NBS.

An inhibition pattern similar to the one obtained manually, was also observed by measuring EST2 residual activity in presence of oxidized pesticides by robotic workstation (Figure 4a). The observed variability within 20% difference, in the automated assay with respect to non-automated measurements, was prevalently related to differences in the incubation times and in the reproducibility of dispensation. Although manual operations have been conducted under controlled conditions, the reproducibility of assays is conditioned by the ability of operator in combination with instrumental errors. In the automated assay the reproducibility of dispensation is assured by the air displacement pipetting of the Hamilton workstation to achieve superior measurement accuracy. In addition, the pipette channels and tips are designed to fit precisely together to eliminate tip distortion and ensure the highest accuracy during liquid handling steps (less of 0.1% error). Moreover, anti-droplet control and liquid level detection technologies safeguard samples and results integrity. Furthermore, the automated assay granted high reproducibility in the time of incubation and measuring, because the system is software supervised and all processes of dispensation, incubation and assay, are scheduled. Also, the automated process allowed to easily collect data at different times in order to obtain a complete inhibition kinetic for each inhibitor.

In agreement with the irreversible inhibitory action of the enzyme catalytic-site by pesticides, we observed a time dependent inactivation of EST2 activity (Figure 4b). Differently from the fast inhibition of EST2 activity in presence of paraoxon, we observed a slowed rate of inhibition for some NBS-treated thio-OPs, requiring from seconds to a few minutes for the complete inhibition of enzyme activity. These differences could also explain the different results obtained in the non-automated assay. A delay in the assay condition due to the manual operations could give different results in the determination of the residual activity at some time resulting in the lack of synchronization between time and residual activity, reducing the accuracy of the determination.

The time dependent inactivation for each thio-OP cannot extensively describe the EST2 ability to covalently bind these compounds, several parameters can be determined, such as the affinity constants. Thus, this is the start point for a future biochemical characterization of these group of pesticides that in this work we have demonstrated can inhibit the EST2 activity.

### 3.5. Pesticide Pseudo Finger-Print

**Figure 5:**
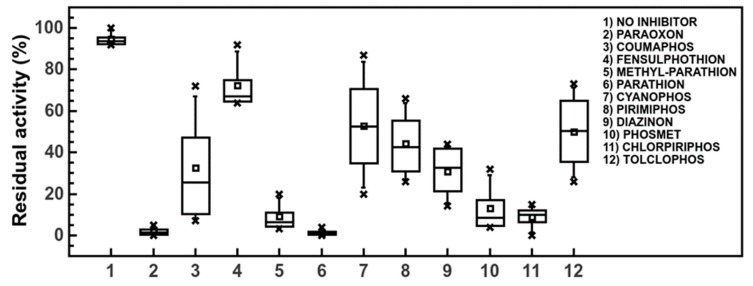
Box-plot of EST2 residual activity in presence of different NBS-oxidized pesticides measured in the range of time from 0 to 9 min by the robotic workstation.

The extension of the incubation time of EST2 in presence of oxidized pesticides, revealed differences in the shape of the curves for the residual activity decrease, indicating a distinctive inhibition kinetics for the different pesticides (Figure 4b). These differences in the inhibition efficiency are probably related to steric hindrance of different OP molecules, as previously demonstrated for some of them [28]. The differences in the inhibition kinetics could be used for the characterization and identifying of pesticides. In Figure 5, a box-plot of EST2 residual activity in presence of NBS-oxidized pesticides measured by the robotic workstation, is illustrated. Except for paraoxon and parathion, almost all the pesticides show a distinctive distribution of the residual activity permitting a pseudo-fingerprinting of compounds. The identification is still limited to the presence of a single pesticide, but the development of mathematical models in data processing software will help in circumventing such limitation by identifying the key compound features. In addition, the development of multi-enzymatic devices, carrying bioreceptors with different specificity towards OP compounds, such as EST2 mutants [29], could provide supplementary parameters in order to discriminate between the different pesticides present in a complex solution more similar to real food samples. Moreover, being able to obtain, by the automated approach, lot of data in a very short time, the use of machine learning or deep learning methods for pesticide recognition could be hypothesized. In fact, these techniques usually based on artificial neural networks, require a large amount of data both to be used as “training” data and data for algorithm validation, in order to perform a task, for example, to predict the pesticide composition in a sample.

## 4. Conclusions

Currently the most widely used methods for the detection of pesticides and similar compounds, still rely on GC- and LC-MS techniques. However, these approaches have some limitations, especially in the integration with the gerneration activities, which require low cost and high speed analysis. In this scenario, biosensors could represent a future breakthrough in the detection of OP pesticides. In fact, biosensors could be used as a preliminary step for the early stage identification or on-site monitoring of OPs in food and environmental samples. In this way, it would become possible to increase the number of analysed samples and focus the most expensive techniques of investigation on a limited number of samples. Moreover, pesticides released into the environment are transformed via series of chemical and biological reactions that often produce unidentified intermediate compounds, some of them being persistent for years or decades in the environment. Tailored biosensing devices will enable the detection for the most part of these molecules, also without reference tests, improving the detection system of these breakdown products. In addition, we are now in the development process for a dual-enzymatic system, suited to in situ oxidation of such compounds by promiscuous artificial and natural peroxidases [34,35], thus avoiding any need for sample pretreatment and possible negative effects on EST2 activity by chemical oxidation. The automation in biosensing detection of pesticides using a robotic system can be easily integrated in industrial production lines, improving the monitoring efficiency, as well as the use of real-time biosensing devices for environmental detection [29]. In this study we have demonstrated the potential for the use of EST2 for the detection of the OP compounds and the possibility to obtain a pseudo finger print for their qualitative detection. Further research must be implemented to obtain a commercially available biosensing device for the OP detection, as the results indicate future possibility of both on-line and off-line bio-assays.

## Supplementary Materials

The following are available online at https://www.mdpi.com/1424-8220/20/5/1365/s1, Table S1: MRM Transitions and Mass Spectrometry Settings; Figure S1: Plot of EST2 residual activity in presence of increasing NBS concentrations; Figure S2: Calibration curve of fluorescence intensity at increasing concentrations of 4-MU in HEPES buffer, measured using the robotic workstation; Figure S3: Calibration curve of inhibitory activity at increasing concentration levels of Paraoxon in HEPES buffer in presence of enzyme, measured using the robotic workstation.
