# Supplementary material for: Highly Sensitive Detection of Chemically Modified Thio-Organophosphates by an Enzymatic Biosensing Device: An Automated Robotic Approach
Source: Sensors (Basel). 2020 Mar 2;20(5):1365. doi: 10.3390/s20051365 (PMC7085774; doi:10.3390/s20051365)
Supplement: Supplementary file 1 [file sensors-20-01365-s001.pdf]

Highly sensitive detection of chemically modified thio-organophosphates by an enzymatic biosensing device: an automated robotic approach.

Giovanni Paolo Cetrangolo[1‡], Janis Rusko[1,2‡], Carla Gori[1], Paola Carullo[1], Giuseppe Manco[1*], Marco Chino[3§], Ferdinando Febbraio[1§*]

[1] Institute of Biochemistry and Cellular Biology – National Research Council (CNR), Via P. Castellino 111 80131 Naples, Italy

[2] Institute of Food Safety, Animal Health and Environment "BIOR", Lejupes street 3, LV-1076 - Riga, Latvia

[3] Department of Chemical Sciences, - University of Naples "Federico II". Via Cintia, 80126 Napoli, Italy

* Correspondence: ferdinando.febbraio@cnr.it +39 081 6132 611 (F.F.); giuseppe.manco@cnr.it +39 081 6132 296 (G.M.)

‡ These authors contributed equally to manuscript (co-first authors).

§ These authors contributed equally to manuscript (co-last authors).

Table of contents

Supplementary Table S1

Supplementary Figure S1

Supplementary Figure S2

Supplementary Figure S3

Supplementary Table S1. MRM Transitions and Mass Spectrometry Settings

Analyte	RT (min)	Q1 (Da)	Q2 (Da)	DP (V)	EP (V)	CE (V)	CXP (V)	Reference
Paraoxon - methyl 1	7.2	248.1	90.1	71	10	37	16	1
Paraoxon - methyl 2	7.2	248.1	202.1	71	10	27	10	1
Paraoxon - ethyl 1	7.7	276.1	220.0	69	10	19	6	2
Paraoxon - ethyl 2	7.7	276.1	248.1	69	10	13	6	2
Phosmet 1	8.1	318.0	160.0	61	10	17	10	2
Phosmet 2	8.1	318.0	133.0	61	10	49	11	2
Parathion - methyl 1	8.2	264.0	125.0	85	10	25	8	3
Parathion - ethyl 1	9.0	292.0	236.0	80	10	20	7	2
Parathion - ethyl 2	9.0	292.0	264.0	80	10	15	7	2
Coumaphos 1	9.2	363.0	227.0	100	10	36	10	2
Coumaphos 2	9.2	363.0	307.0	100	10	25	10	2
Tolclofos - methyl 1	9.7	301.0	268.9	59	10	23	6	2
Tolclofos - methyl 2	9.8	301.0	175.0	59	10	35	6	2
Diazinon 1	10.0	305.0	169.0	80	10	27	11	2
Diazinon 2	10.0	305.0	153.0	80	10	28	11	2
Pirimiphos - methyl 1	10.4	306.1	164.1	75	10	29	6	2
Pirimiphos - methyl 2	10.4	306.1	108.0	75	10	40	6	2
Chlorpyrifos 1	10.8	350.0	198.0	82	10	29	9	2

Chlorpyrifos 2	10.8	350.0	97.0	82	10	49	9	2

References:

(1) Fillâtre, Y.; Rondeau, D.; Daguin, A.; Jadas-Hecart, A.; Communal, P.-Y. Multiresidue Determination of 256 Pesticides in Lavandin Essential Oil by LC/ESI/SSRM: Advantages and Drawbacks of a Sampling Method Involving Evaporation under Nitrogen. *Anal. Bioanal. Chem.* **2014**, *406* (5), 1541–1550.

(2) Wang, J.; He, Z.; Wang, L.; Xu, Y.; Peng, Y.; Liu, X. Automatic Single-Step Quick, Easy, Cheap, Effective, Rugged and Safe Sample Preparation Devices for Analysis of Pesticide Residues in Foods. *J. Chromatogr. A* **2017**, *1521*, 10–18.

(3) Feng, X.; He, Z.; Wang, L.; Peng, Y.; Luo, M.; Liu, X. Multiresidue Analysis of 36 Pesticides in Soil Using a Modified Quick, Easy, Cheap, Effective, Rugged, and Safe Method by Liquid Chromatography with Tandem Quadruple Linear Ion Trap Mass Spectrometry. *J. Sep. Sci.* **2015**, *38* (17), 3047–3054.

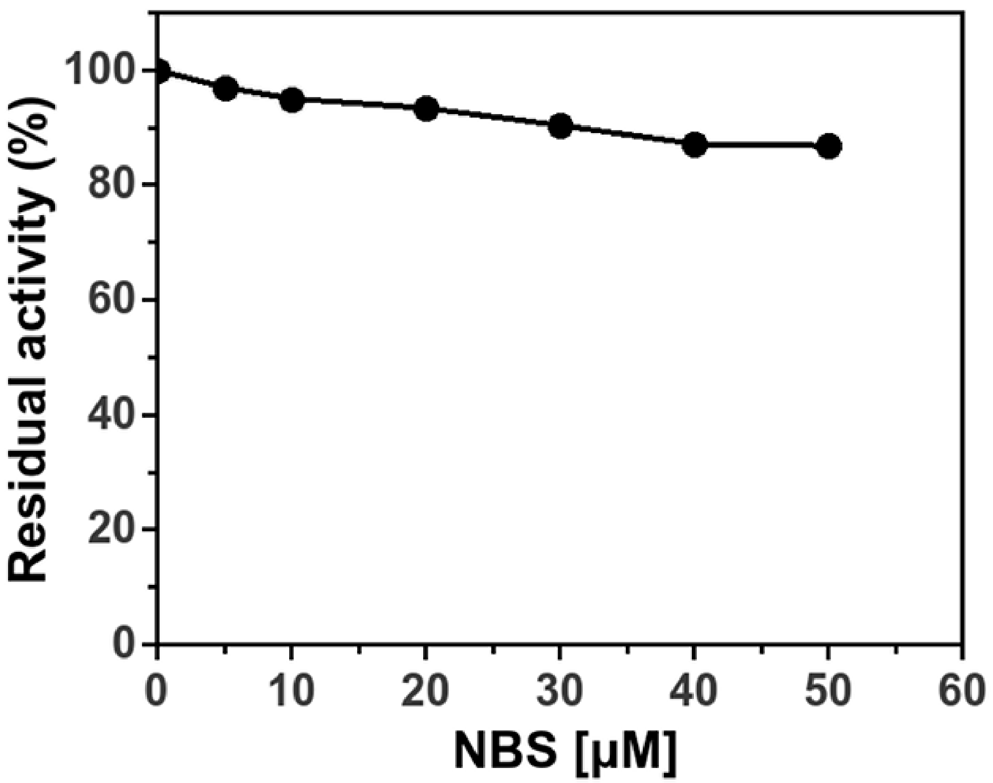

Supplementary Figure S1. Plot of EST2 residual activity in presence of increasing NBS concentration levels.

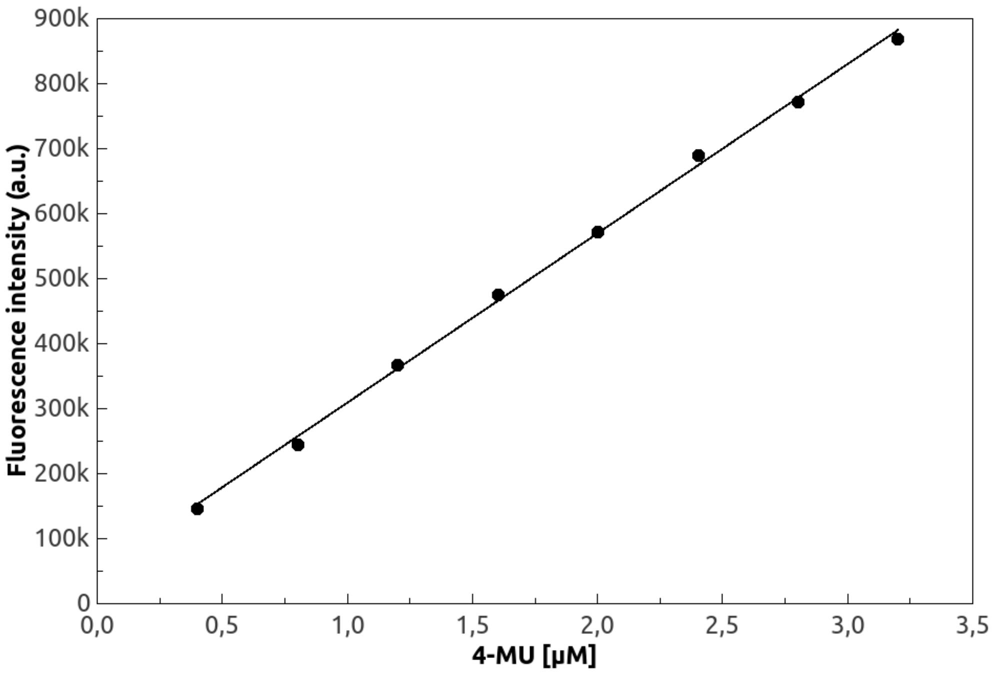

Supplementary Figure S2. Calibration curve of fluorescence intensity at increasing concentration levels of 4-MU in HEPES buffer, measured using the robotic workstation.

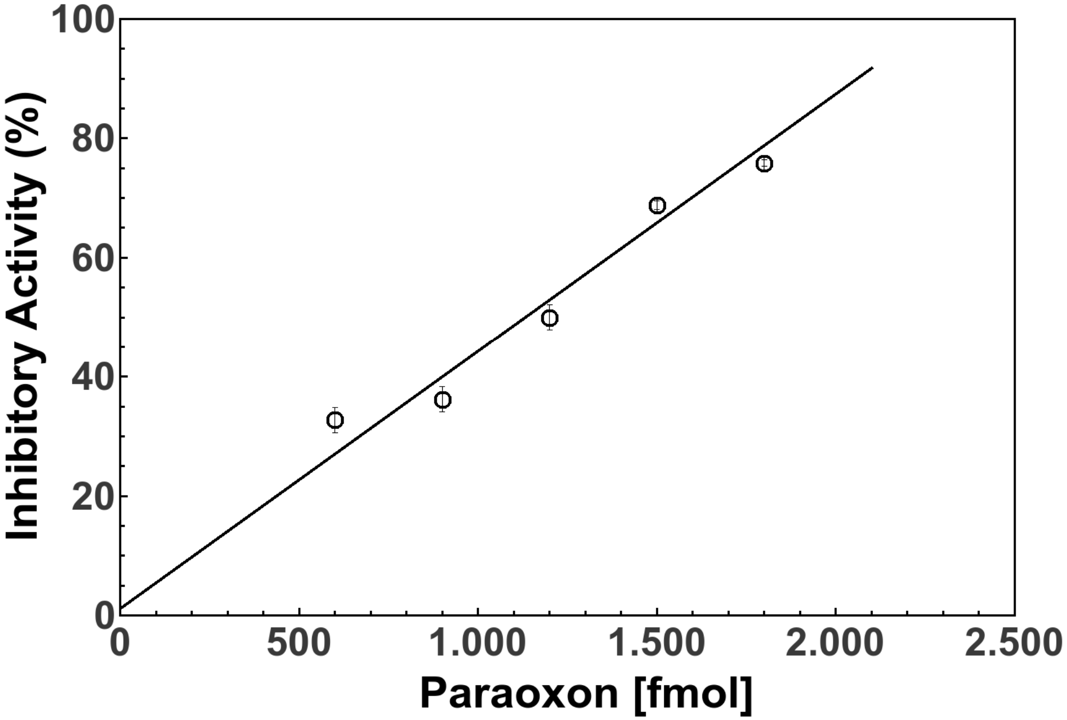

Supplementary Figure S3. Calibration curve of inhibitory activity at increasing concentration levels of Paraoxon in HEPES buffer in presence of enzy
